# Aging and Down Syndrome

**DOI:** 10.1155/2012/412536

**Published:** 2012-07-11

**Authors:** Elizabeth Head, Wayne Silverman, David Patterson, Ira T. Lott

**Affiliations:** ^1^Department of Molecular and Biomedical Pharmacology, Sanders-Brown Center on Aging, University of Kentucky, 800 South Limestone Street, Lexington, KY 40536, USA; ^2^Kennedy Krieger Institute, Johns Hopkins University School of Medicine, 707 N. Broadway, Baltimore, MD 21205, USA; ^3^Eleanor Roosevelt Institute and the Department of Biological Sciences, University of Denver, 2101 E. Wesley Avenue, Denver, CO 80208, USA; ^4^Department of Pediatrics and Neurology, School of Medicine, University of California-Irvine (UCI), 101 The City Dr. ZC 4482, Orange, CA 92868, USA

## 1. Introduction

The initial clinical description of Down syndrome (DS) was made by Down in 1866 [1] and identified as trisomy of chromosome 21 by Lejeune et al. in 1959 [2]. DS, or trisomy 21, is one of the most common causes of intellectual disability (ID), and recent national prevalence estimates suggest that 14.47 per 10,000 live births are infants with DS, leading to an average of 6037 annual DS births [3]. This represents an increase from previous prevalence rates. Characteristic physical features, deficits in the immune and endocrine systems, and delayed cognitive development [4] can be present in children with DS. 

Improvements in medical care for children and adults with DS have led to significant extensions in lifespan and enhanced quality of life. As a consequence, up to 35 years of age, mortality rates are comparable in adults with DS to individuals with ID from other causes. However, after age 35, mortality rates double every 6.4 years in DS, as compared to 9.6 years for people without DS [5], and the currently estimated life expectancy of a 1-year-old child with DS is between 43 and 55 years (depending on the level of disability). Although longevity in adults with DS has been increasing progressively, these increases have been substantially lower for some minority groups [6, 7]. Further, as described by L. Thorpe et al., adults with DS are still disadvantaged compared to adults with other types of ID in terms of mortality rate, with multiple comorbidities being of significant concern (e.g., depression, seizures). Significant contributors to quality of life in aging individuals with DS also include gait disturbances (B. A. Smith et al.) and ophthalmic disorders (e.g., S. J. Krinsky-MC. Hale et al.), both of which increase with age. 

## 2. Dementia and Aging in DS

A key concern to aging adults with DS is the increasing risk for developing Alzheimer's disease (AD). The profile and sequence of cognitive impairments in adults with DS are similar to those seen with AD in the general population [8–13]. Memory processes are affected early in the course of DS dementia [14]. Severe cognitive deterioration, such as acquired apraxia and agnosia, has been reported in 28% of individuals with DS at age 30 years, with a higher prevalence of these impairments in the subsequent years [8, 9]. The earliest manifestations of dementia in DS may involve changes in personality and behavior [15–17]. Pragnosia or socially deficient communication may be an early sign of frontal lobe dysfunction in DS and can represent a striking change from previous well-developed social capacities [18]. Individuals with DS typically show less of a decline in language compared to performance skills associated with aging throughout adulthood as compared to individuals with ID but not DS [19]. The diagnosis of dementia in DS can be challenging against the background of pre-existing intellectual impairment. Standardized criteria for the diagnosis of dementia in DS include both informant-based and direct measures [16, 20–22]. The severity of preexisting cognitive impairment may also be a predictor of the rate of cognitive deterioration in DS [23]. 

Potentially modifiable risk factors are now being identified that can lead to enhanced susceptibility to dementia in people with DS [24]. For example, women with DS may develop dementia 10–20 years earlier than women in the general population [9, 25, 26]. One possible mechanism underlying increasing dementia risk for women with DS is suggested by J. H. Lee et al. The study describes a genetic polymorphism in the hydroxysteroid-17*β*-dehydrogenase gene that is responsible for converting estrone to estradiol. Polymorphisms of this gene may lead to changes in activity of hydroxysteroid-17*β*-dehydrogenase and modify circulating levels of neuroprotective estrogen. In a cohort of women with DS followed longitudinally, the onset of dementia was linked to three of five single nucleotide polymorphisms (SNPs) in this gene, and women with high-risk SNPs were 2-3 times more likely to develop AD. 

## 3. Neurobiology of Aging in DS

Middle-aged individuals with DS develop AD pathology [25, 27–29], no doubt contributing to their high risk of developing dementia [9, 30]. Still, not every individual with DS will develop dementia. Although clinical signs of dementia are more commonly observed when individuals are over 50 years of age [9, 31–33], by 40 years of age, virtually all individuals with DS have neuropathological changes that are consistent with AD, including senile plaques and neurofibrillary tangles (NFT) [13, 27, 28]. Senile plaques contain the beta-amyloid peptide (A*β*) toxic to neurons and thought to be a causative event in the pathogenesis of AD [34, 35]. 

DS involves the overexpression of the amyloid precursor protein (APP) on chromosome 21. APP is cleaved sequentially by beta- and gamma-secretases to release A*β*, which forms toxic conformations and aggregates (e.g., senile plaques) in the AD and DS brain [34]. Indeed, the development of AD neuropathology in most individuals with DS after the age of 40 years is considered to be key evidence in support of the amyloid cascade hypothesis as a cause for sporadic AD. Beta-secretase has been characterized as the enzyme beta-amyloid cleaving enzyme (BACE), of which a homologous version, BACE2, is present on chromosome 21 [36]. However, despite increases in BACE2 mRNA in DS brain, protein levels appear similar in DS compared to non-DS brain. Further, BACE2 activity appears to decrease the production of A*β* from APP in contrast to the activity of BACE1. R. L. Webb and M. P. Murphy also describe evidence that BACE activity overall is not increased in the aged DS brain, leading to the conclusion that APP overexpression may be the prime cause of A*β* overproduction. It is also fascinating to consider that despite earlier ages of onset of A*β* deposition in the brain (~30 years), people with DS are able to compensate for progressive AD neuropathology and many people with DS do not show signs of cognitive decline until their 50s or even later [24]. Similarly, there are people without DS who by imaging studies or autopsy examinations show A*β* deposition in the brain but are clinically normal, suggesting that similar compensatory processes may also occur [37–39].

A novel hypothesis is also provided by A. Reed-Cossairt et al. regarding a role for reduced clearance of A*β* as a consequence of slower cerebrospinal fluid (CSF) turnover. In addition, vascular dysfunction, specifically in the jugular reflux, may be a significant contributor to slowed CSF turnover and the development of dementia in adults with DS. An additional consequence of reduced vascular function may be the development of white matter neuropathology that is typically seen in AD. Many gaps in our knowledge regarding this potential feature of aging in DS that could be modified with appropriate interventions are also discussed by Reed-Cossairt. 

Additional neurobiological events that may impact the risk of dementia may also compromise cognition in DS. As summarized by J. P. Lockrow et al., a loss of neurons in the locus coeruleus and basal forebrain (BFCNs) can lead to reductions in two neurotransmitters that play a critical role in learning and memory, norepinephrine, and acetylcholine. Further, these authors provide additional data suggesting that reduced norepinephrine may also lead to increased neuroinflammation and degeneration in the hippocampus (another area critical for memory). Reduced neurotransmitter levels in DS may also lead to a loss of trophic support for neurons in the DS brain with age. Based upon research in mouse models of DS (described in detail by G. N. Vacano et al.), a loss of brain derived neurotrophic factor (BDNF) occurs in response to norepinephrine losses and leads to cognitive deficits. A decrease in BDNF, in turn, has been linked to enhanced vulnerability of neurons to oxidative stress, suggesting a cycle of increasing insults that may eventually lead to neurodegeneration or death.

Oxidative damage has been extensively studied in people with DS, because a significant number (>10) of genes encoding proteins relevant to oxidative damage [40–42] and ROS production located on chromosome 21 [43]. Many of these are overexpressed in DS. SOD1 has been perhaps the most studied protein with regard to ROS metabolism in DS. Increased levels of this endogenous antioxidant without parallel increases in catalase can lead to higher levels of hydrogen peroxide. As reviewed by M. Perluigi et al., oxidative damage may be a significant contributor to neurodegeneration associated with the AD neuropathology seen in DS with advancing age. Specifically, in the aging DS brain, the presence of age-associated A*β* can in turn cause oxidative damage, but there is also evidence that compensatory mechanisms may support normal neuronal function until some threshold is crossed. Further, changes in mitochondrial functioning may produce damaging free radicals that contribute to oxidative stress, given that we see higher levels of mitochondrial DNA mutations in DS (P. E. Coskun and J. Busciglio), although the combination of mitochondrial dysfunction and oxidative stress may lead to adaptive responses in DS, perhaps prior to the development of AD.

A*β* and oxidative damage may contribute to brain inflammation, either independently or in concert. Neuroinflammation, however, has not been studied as extensively as these two other markers of neuropathology and represents an area of focus that may be highly relevant to aging in DS (D. M. Wilcock) [44, 45]. Given that genes involved with neuroinflammation are located on chromosome 21, the neuroinflammatory milieu in DS may be different from AD in the general population. For example, S100*β* is present in triplicate in DS, is expressed by astrocytes, and is released in response to inflammatory cytokines. As shown in Table 1 of the review by D. M. Wilcock, this is one of multiple genes that could lead to enhanced neuroinflammation in DS, although these genes may also prime the brain towards an M1 inflammatory response. Inflammatory responses may lead to enhanced vulnerability of DS neurons in the presence of A*β*—tangles and oxidative damage—and could be a significant target for intervention. Neuroinflammation has not been fully explored as a function of age in DS and is an active area of research in AD in the general population.

G. Tansley et al. provide novel evidence that circulating levels of 24S-OH-cholesterol, thought to reflect brain levels of cholesterol, are unchanged in aging individuals with DS compared to those without DS. Further, the overall lipid metabolism profile of plasma observed in DS is similar to those without DS. The exception may be brassicasterol levels, which are reduced for older DS individuals compared to those without DS. While this is a very intriguing finding and may reflect AD-associated neuropathology, this is the first report of the effect and further confirmation is required. 

## 4. Pharmacological and Nonpharmacological Intervention Strategies

There are only 5 FDA-approved drugs for the treatment of AD in the general population, and these have met with moderate or little success for the treatment of AD in DS [46–51]. This suggests that other disease-modifying approaches are going to be critically important for future therapeutics. Prevention, however, may be the most promising approach to healthy aging in DS and may include both pharmacological and nonpharmacological interventions.

Critically important to the development of novel therapeutics or prevention strategies is the use of mouse models for DS where promising strategies can be first tested. There are several mouse models for DS that capture developmental and aging-associated phenotypes (G. N. Vacano et al.). Although DS is a complex genetic disorder, careful dissection of the role of individual or groups of genes to the DS phenotype provides an exciting approach for the development of new interventions. In combination with existing mouse models for AD, promising new pharmacological or nonpharmacological treatments may be identified. However, it is also critical to note that translation of outcomes from mouse studies to human clinical trials is not necessarily direct, but the mouse studies provide important proof of principle outcomes that can be pursued. 

As one example of using this approach, J. P. Lockrow et al. review studies suggesting that the norepinephrine precursor L-threo-DOPS (Droxidopa) can improve learning and memory in DS mice and may be a possible target for DS clinical trials addressing improvement of age- and AD-associated cognitive dysfunction. As a parallel component to enhancing norepinephrine function, neuroinflammation appears to be intimately linked to the levels of this neurotransmitter and increased in DS aging mice. 

Another pharmacological approach to preventing AD in people with DS may be to modify the production of A*β* due to overexpression of APP (R. L. Webb and M. P. Murphy). Clinical trials are currently addressing the reduction of BACE activity in sporadic cases of AD (http://www.clinicaltrials.gov/), but gamma-secretase inhibitors may not be a viable option given adverse side effects. An intriguing report in a mouse model of DS showed that reducing BACE activity in young animals reduced learning and memory deficits, suggesting that APP overexpression and production of A*β* may not only be involved with AD development with age but also contribute to intellectual disability in younger individuals [52].

Reducing oxidative damage (which is a lifelong issue for DS) may require a multitargeted approach that is preventative in nature, given that supplementing demented adults with DS with antioxidants (or individuals with AD in the absence of DS) has shown little or no benefit to clinical outcomes [53]. Indeed, compensatory mechanisms at younger ages in DS may be enhanced by antioxidant supplementation (M. Perluigi and D. A. Butterfield). Focusing on mitochondrial dysfunction is also a promising approach, as these organelles are the primary producers of reactive oxygen species (P. E. Coskun and J. Busciglio). In addition, isolated mitochondria have a higher rate of DNA mutations, suggesting a progressive exacerbation in mitochondrial function in DS. Given this evidence for mitochondrial dysfunction in DS, there are several possible interventions that may reduce age-associated declines (e.g., dietary changes and/or supplementation with mitochondrial cofactors). A combinatorial approach may be particularly valuable for adults with DS who may benefit from a supplement including both antioxidants (e.g. vitamins E and C) and mitochondrial co-factors (e.g., lipoic acid, acetylcarnitine). However, it may be critical to use these approaches as a preventative measure rather than as a treatment protocol for AD in DS [53]. Given the interaction between A*β*, oxidative damage and neuroinflammation, and the relative paucity of data regarding inflammation in the aging DS brain, unexplored potential new targets for intervention may exist (D. M. Wilcock).

Several key issues highlighted in this special issue topics suggest that lifestyle modification and regular health monitoring may also lead to successful aging in people with DS. For example, although older adults show decreased stability and efficiency in gait during walking, evidence for adaptation suggests potential for improvements with appropriate interventions (B. A. Smith et al.). Changes in gait should be taken into consideration, as they may lead to less physical activity and/or functional decline. 

A substantial number of people with DS develop ophthalmic disorders, affecting up to 50% of adults between 50 and 59 years of age (S. J. Krinsky-MC. Hale et al.). The development of age-associated visual deficits occurs at younger ages in DS than that in the general population. The presence of ophthalmic disorders is higher in DS individuals with more severe intellectual disability, leading to additional challenges and significant functional consequences. Interestingly, in S. J. Krinsky-MC. Hale's report of longitudinally followed individuals with DS, cataracts were the most frequent problem in older adults but were not associated with the level of ID. However, the presence of cataracts does compromise functioning and readily available treatments can improve quality of life for affected individuals with DS.

Diet may be a very important area to consider for healthy aging in DS. For example, the reduced levels of brassicasterol reported by G. Tansley et al. may be modifiable by diet. Whether this would mechanistically reduce the development of AD or impact on longitudinal changes in cognition has yet to be determined. Additionally, managing estrogen levels in aging women with DS may help to reduce their risk for developing AD dementia, but an individualized approach including genetic risk as determined by the presence of SNPs on the HSD17B1 gene may need to be incorporated (J. H. Lee et al.).

## 5. Summary

This special issue of the *Current Gerontology and Geriatrics Research* journal was intended to cover selected issues defining the concerns faced by adults with Down syndrome as they grow older. As can be seen from this selection of papers, substantial gaps in our knowledge of the aging process in people with DS continue to persist and are critically important to address. Further, approaches for maintaining healthy aging in individuals with DS may also inform strategies for enhancing quality of life for other adults with ID or indeed in the general population. Ongoing longitudinal studies that monitor changes in health status, cognition, function, the development of dementia, and mortality will all be critically important for informing the development of policies and interventions to promote healthy aging in DS. A key challenge to aging adults with DS is the increasing risk for developing dementia; yet our social and medical infrastructure is not as well prepared for providing care to adults with DS as they age relative to the outstanding support available to children with DS and their families. The use of FDA-approved treatments for AD in the general population has met with limited success in people with DS [46]. Therefore, it is critically important to explore novel interventions and potential prophylactic approaches that may provide individuals with DS the best possible opportunity to age gracefully. Such interventions along with further study of aging, dementia, and Alzheimer disease in DS are likely to be of fundamental importance in understanding AD in the general population. 
